# COVID-19 Is Further Subdivided Into COVIP-19 and COVII-19

**DOI:** 10.3389/fpubh.2020.00301

**Published:** 2020-06-16

**Authors:** Shiliang Song

**Affiliations:** Department of Internal Medicine, Taicang Hospital Affiliated to Suzhou University, Suzhou, China

**Keywords:** COVID-19, coronavirus pneumonia, coronavirus infection, disease nomenclature, classification of disease, disease diagnosis, compiling textbooks, economic and financial crisis

In less than half a year, COVID-19 has swept the world, severely threatened the safety of all mankind, and caused great social panic and global economic and financial crisis. The WHO officially named the disease caused by SARS-CoV-2 as COVID-19 on February 11, 2020 [in COVID-19, CO stands for corona, VI stands for virus, and D stands for disease (1)]. This is authoritative and easy to read and remember and has been accepted all over the world, but there is a small disadvantage that the name is relatively general. After all, COVID-19 is caused by SARS-CoV-2 and mainly invades the human respiratory system, and some cases of the disease are characterized by extensive lung inflammation (2, 3). In these cases, autopsy reports reveal that COVID-19 mainly attacks the lungs (4). Therefore, it is necessary to highlight the term pneumonia. It is also important to consider other patients with SARS-CoV-2 invasion whose clinical symptoms and signs are mild or who are even asymptomatic infection, and for whom imaging examination does not show the characteristic changes of pneumonia (2–5). X-ray or CT imaging features of COVIP-19: Multiple small patches and pulmonary interstitial changes occur in the early stage, obvious in the outer zone of the lung, and develop into multiple ground-glass opacities and infiltrates in the bilateral lung. Pulmonary consolidation may appear in severe cases) (6). I therefore further divide COVID-19 into two types, COVIP-19 and COVII-19, namely, 2019 coronavirus pneumonia and 2019 coronavirus infection (In COVIP-19, CO stands for corona, VI stands for virus, and *P* stands for pneumonia; in COVII-19, CO stands for corona, VI stands for virus, and I stands for infection). In the future, with the increase in the number of clinical and pathological reports on COVID-19 and the increase in autopsy reports, it will be possible to determine whether further classification is required based on the different organs affected by SARS-CoV-2; this requires further research. However, regardless of whether it is further subdivided, COVIP-19 is the most important variation, just as pulmonary tuberculosis is most important in the classification of tuberculosis (7). Health Industry Standard of the People's Republic of China WS196-2017. Classification of tuberculosis. According to location of pathological changes: (1) Pulmonary tuberculosis: points to pathological changes happening in the lung, trachea, bronchus, and pleura, etc. (2) Extrapulmonary tuberculosis: refers to tuberculosis in the lungs outside the organs and parts. Named according to the diseased organ and location, such as intestinal tuberculosis, renal tuberculosis, lymphoid tuberculosis, tuberculous peritonitis, tuberculous meningitis, tuberculosis of bone and joint, etc.) (7).

It is recommended that in the future, all countries reporting COVID-19 should report COVIP-19 and COVII-19 separately. At the same time, it is suggested that when summarizing the daily COVID-19 data, websites such as the WHO, Johns Hopkins University, and Worldometer display the number of cases in two columns, COVIP-19 and COVII-19, respectively, or only list the case numbers for COVIP-19. The number of cases reported in this way will be greatly reduced and this may greatly ease the tension and anxiety of people around the world, while also benefiting the world economy and stabilizing the financial markets. Since respiratory failure caused by COVIP-19 is the main cause of death in this disease (2–4), we can focus on the treatment of COVIP-19 and reposition medical resources. It is also necessary to quarantine COVII-19 patients for about 14 days because it is also infectious, and isolation can only be terminated if all secondary nucleic acid tests are negative (6).

The recommendation for clinicians is: “suspected COVID-19 or COVID-19 patient” (abbreviated as COVID-19?) can be used as an initial diagnosis in the outpatient setting. However, a definitive diagnosis should be made by using the results of nucleic acid detection and lung CT or X-ray imaging to decide on COVIP-19 or COVII-19. My classification method not only follows the WHO nomenclature of COVID-19 but also refines the exact definition of COVID-19, which will help clinicians to accurately diagnose the disease.

On February 8, 2020, the National Health Commission of China referred to COVID-19 as novel coronavirus pneumonia, abbreviated as NCP, and on February 21, 2020, the English abbreviation was changed to COVID-19 (8). It is a little inaccurate, as the two names COVID-19 and novel coronavirus pneumonia do not have a one-to-one correspondence. According to the typing method described by the author of this article, COVIP-19 and novel coronavirus pneumonia are well-corresponding and unified.

In the future, when compiling textbooks (9), the chapter for COVID-19 should be divided into two sections, COVIP-19 and COVII-19.

## Author Contributions

The author confirms being the sole contributor of this work and has approved it for publication.

## Conflict of Interest

The author declares that the research was conducted in the absence of any commercial or financial relationships that could be construed as a potential conflict of interest.
